# Condensed low-dose total skin electron beam therapy for mycosis fungoides: an institutional retrospective review and subgroup analysis of patients with large cell transformation

**DOI:** 10.1007/s00403-025-04030-3

**Published:** 2025-03-08

**Authors:** Louis Cappelli, Megan Cappelli, Nilanjan Haldar, Tiara Paul, Jenna Mandel, Tingting Zhan, Neda Nikbakht, Wenyin Shi

**Affiliations:** 1 Department of Radiation Oncology, Thomas Jefferson University Hospital, Philadelphia, PA USA; 2 Department of Dermatology, Thomas Jefferson University Hospital, Philadelphia, PA USA; 3 Sidney Kimmel Medical College, Thomas Jefferson University Hospital, Philadelphia, PA USA; 4 Department of Pharmacology, Physiology and Cancer Biology, Thomas Jefferson University Hospital, Philadelphia, PA USA; 5 Department of Radiation Oncology, Thomas Jefferson University Hospital, Sidney Kimmel Cancer Center, 111 St 11th Street, Philadelphia, Pennsylvania 19107 USA

**Keywords:** Mycosis fungoides, Large cell transformation, Total skin electron beam therapy, patient reported outcomes, survival

## Abstract

Low-dose total skin electron beam therapy (TSEBT) is an effective treatment option for mycosis fungoides (MF) with proven palliative effects and reduced toxicity. Presented is an institutional analysis of survival/response rate and quality of life for MF patients with subgroup analysis of those possessing pathologic large cell transformation (LCT). This is a single institutional retrospective review of patients with mycosis fungicides treated from 2014 to 2023 with low-dose TSEBT. All patients received 12 Gy in 6 fractions every other day with the modified Stanford technique, with boosts to shadowed sites between treatments, completed in 2 weeks. Outcomes evaluated included clinical response, duration of and time to response, patient-reported quality of life, and physician-scored disease burden. Forty-six patients were included in the study, 28 male and 18 female, with a median age 66.5 (range 32.7–90.6). Stage IB was most common at the time of TSEBT (41.3%). Median follow up was 44.5 months. The overall response rate was 91.3% (52.2% partial response, 19.6% complete and near complete response). The median duration of response was 8.2 months (range, 6.1–28.7), and the median time to best response was 3.5 months (range, 2.7–5.6). Quality of life (QOL) and disease burden continued to show significant benefit after TSEBT (*p*<0.001). In a subgroup analysis, 18 patients (39.1%) were found to have large cell transformation (LCT) at diagnosis. LCT was associated with higher presenting stage prior to TSEBT (*p*=0.016) and a better response to treatment (*p*=0.040). However, median duration of response was only 7.4 months in the patients with LCT vs. 39.4 months in the patients without (*p*=0.003). Condensed Low-dose TSEBT is a convenient treatment with favorable clinical outcomes and low toxicities in patients with mycosis fungoides. Patients with LCT may have shorter duration of treatment response. Further studies are warranted to validate this finding.

## Introduction

Mycosis fungoides (MF) is the most common form of primary cutaneous T-cell lymphomas in the United States of America, with an estimated incidence of 1 in 100,000 individuals [1, 2]. MF is a radiosensitive neoplasm, total skin electron beam therapy (TSEBT) is often used for patients with generalized skin involvement with excellent clinical response and control of their disease [1, 3]. MF is currently staged following recommendations from the International Society for Cutaneous Lymphoma (ISCL) and the cutaneous lymphoma task group of the European Organization for Research and Treatment of Cancer (EORTC) utilizing the TNMB system [4]. There is, however, a growing interest in integrating other prognostic factors into the current staging system as recent research has better characterized the entity. One such factor that may be associated with poor response rates is large cell transformation (LCT), defined as the presence of cells at least four times greater than the size of a small lymphocyte within the diagnostic skin biopsy sample [5].

TSEBT was initially developed at Standford University in the 1960s with doses of 30–36 Gy that are associated with improved complete response rates [6]. Despite TSEBT being one of the most effective therapies for early-stage and advanced MF in terms of response and disease control, it is associated with significant toxicity, including skin xerosis, alopecia, and edema [3]. Regardless of this, most patients treated with high-dose TSEBT (HD-TSEBT) will eventually recur with options for re-irradiation limited due to elevated risk for debilitating radiation-induced toxicities, including anhidrosis. Multiple studies have been conducted evaluating the application of low-dose TSEBT (LD-TSEBT) at regimens of 10–12 Gy and have shown clinically meaningful response rates with similar duration in response and a more favorable toxicity profile compared to HD-TSEBT [1, 3]. LD-TSEBT also has the added advantage of allowing patients to receive further radiation therapy following recurrence with a lower risk for treatment-induced toxicity with re-irradiation. Due to the advantages of LD-TSEBT, institutions such as the UK Cutaneous Lymphoma Group and the EORTC now consider LD-TSEBT as a favored option for patients presenting diffuse skin involvement as compared to HD-TSEBT [7, 8].

Currently, the efficacy of LD-TSEBT for patients with LCT remains an area of active study. Additionally, the optimal dose and fractionation scheme for LD-TSEBT has yet to be agreed upon. This article presents our institute’s experience and results treating MF patients with and without LCT treated with LD-TSEBT at 12 Gy in six 2 Gy fractions administered every other day.

## Methods

This study was conducted in accordance with and approved by the Thomas Jefferson University Institutional Review Board (IRB). The privacy and confidentiality of all participant information were strictly maintained throughout the research process.

This is a single institutional retrospective review of patients with MF received LD-TSEBT at Thomas Jefferson University from 2014 to 2023. Patient data was collected from medical records.

Patient demographics and characteristics, stage of disease, and the presence of LCT prior to LD-TSEBT were collected. Following the completion of radiation, patient response to treatment, duration of disease response and toxicities were recorded.

Following an MF diagnosis, LCT must be classified histopathologically. LCT is defined by the presence of large cells, characterized by cells at least 4 times the size of small lymphocytes, composing≥25% of the entire infiltrate [9].

### Radiation treatment

All patients received 12 Gy in 6 fractions every other day with the modified Stanford technique, with boosts to shadowed sites at risk between treatments [3]. Shadowed sites included the soles of the feet, scalp, pannus, perineum, and inframammary folds. Boost doses to shadowed sites would include either 8 Gy in 2 fractions and/or 4 Gy in 1 fraction regimens. Patients were positioned standing in a specially designed TSEBT box with a source-to-skin distance of 3.9 m. The treatment involved six specific positions, using dual fields: upper and lower anterior-posterior/posterior-anterior, right and left anterior oblique, and right and left posterior oblique. Radiation was administered using 6-MeV electrons, targeting the depth of maximum dose (dmax) at a rate of approximately 2400 MU per minute. The field dimensions were 40×40 cm, and the gantry was angled at 270° ± 18° to encompass both the upper and lower fields, with a lucite beam spoiler applied. Dose verification in vivo was carried out at 10 positions using optically stimulated luminescence, along with diodes for boost areas. Lead shielding was used to protect the nailbeds and eyelids [8].

During TSEBT, patients did not receive concomitant systemic therapy for management of their disease.

### Outcomes

Primary outcomes included clinical response and duration of and time to response. Response assessment of the skin was conducted using the modified skin weighted assessment tool (mSWAT). The mSWAT score was calculated prior to treatment and at post-treatment follow-up. Similarly, body surface area (BSA) involvement was collected for patients prior to and following treatment to assess treatment response.

Responses to LD-TSEBT were characterized as complete response (CR), near complete response (nearCR), partial response (PR), and stable disease (SD). A CR was defined as 100% clearance of disease with mSWAT=0. PR was defined as a>50% reduction in mSWAT from baseline, SD was defined as no clinical changes on exam following treatment, and a nearCR was defined as <1% of disease remaining on the body surface area (BSA) following treatment.

Secondary outcomes included metrics of survival including overall survival (OS) at five years, time to best treatment response (TBR), duration of treatment response (DTR), and progression free survival (PFS). TBR was defined as the time from TSEBT treatment to nadir in BSA and mSWAT score as assessed by a dermatologist. DTR was defined as the time that elapsed from the date of TBR until the patient was determined to have had progression of disease (PD). PD, in turn, was defined as an increase in the patients mSWAT score from nadir by an amount greater than or equal to 50% of the patients baseline mSWAT. PFS was defined as the time from LD-TSEBT to PD.

Patient reported quality of life was assessed with Dermatology Life Quality Index (DLQI) for each patient prior to and following treatment with LD-TSEBT. DLQI score were defined by the following: scores of 0–1 indicating the symptoms of disease having no effect on the patient’s life, 2–5 indicating a small effect on the patient’s life, 6–10 indicating a moderate effect on the patient’s life, 11–20 indicating a very large effect on the patient’s life, and 21–30 indicting extremely large effect on the patient’s life.

### Statistics

Statistical analysis was carried out using Kaplan-Meier and log-rank tests were performed to compare survival outcomes between the subgroups of patients with and without LCT. Descriptive statistics were used for calculating mean, median, and range of patient characteristics. Fischer’s Exact tests were used to determine the statistical significance between variables and were performed via R software.

## Results

A total of 46 patients were identified who received condensed LD-TSEBT for the management of CTCL between June 2014 to February 2023. The median age at the start of radiation therapy was 66.5 years (range, 32.7–90.6), 18 (39.1%) were female and 28 (60.9%) were male. Regarding ethnicity, 26 (56.5%) of the patients identified as white, 15 (32.6%) of the patients identified as black, 4 (8.7%) of the patients identified as Hispanic, and 1 (2.2%) patient identified as Asian. Pre-TSEBT, the median mSWAT score was 50, with a range of 5.5-149.5. Prior to treatment, 3 (6.5%) patients were stage IA, 19 (41.3%) were stage IB, 4 (8.7%) were stage IIA, 5 (10.9%) were stage IIB, 8 (17.4%) were stage IIIA, 1 (2.2%) was stage IIIB, 5 (10.9%) were stage IVA, and 1 (2.2%) was stage IVB. The recorded staging was the highest extent of disease prior to initiation of TSEBT, except for the 3 Stage IA patients, which were previously Stage IB, IIB, and IVA. Of the 46 patients included in the study, 18 (39.1%) were identified to have LCT (Table 1).

**Table 1:** Patient characteristics prior to treatment

		All Patients (*n*=46; 100%)	LCT Present (*n*=18; 39.1%)	LCT Absent (*n*=28; 60.9%)
Age (years)	Median (Range)	66.5 (32.7–90.6)	68.8 (47.3–82.4)	64.6 (32.7–90.6)
Sex	M: n (%) F: n (%)	28 (60.9%) 18 (39.1%)	11 (61.1%) 7 (38.9%)	17 (60.7%) 11 (39.3%)
Race	Asian: n (%) Black: n (%) Hispanic: n (%) White: n (%)	1 (2.2%) 15 (32.6%) 4 (8.7%) 26 (56.5%)	0 7 (38.9%) 2 (11.1%) 9 (50.0%)	1 (3.6%) 8 (28.6%) 2 (7.1%) 17 (60.7%)
Mycosis Subtype	Folliculotropic: n (%) Syringotropic: n (%)	22 (47.8%) 19 (41.3%)	11 (61.1%) 10 (55.6%)	11 (39.3%) 9 (32.1%)
Pre-XRT Disease Stage	1 A: n (%) 1B: n (%) 2 A: n (%) 2B: n (%) 3 A: n (%) 3B: n (%) 4 A: n (%) 4B: n (%)	3 (6.5%) 19 (41.3%) 4 (8.7%) 5 (10.9%) 8 (17.4%) 1 (2.2%) 5 (10.9%) 1 (2.2%) * ***p*** **=0.001**	2 (11.1%) 5 (27.8%) 2 (11.1%) 3 (16.7%) 0 1 (5.6%) 5 (27.8%) 0	1 (3.6%) 14 (50.0%) 2 (7.1%) 2 (7.1%) 8 (28.6%) 0 0 1 (3.6%)
Pre-XRT T-Stage n* = 42	1: n (%) 2: n (%) 2a: n (%) 2b: n (%) 3: n (%) 4: n (%)	4 (9.5%) 13 (31.0%) 2 (4.8%) 6 (14.3%) 7 (16.7%) 10 (23.8%) *p=0.121	3 (18.8%) 4 (25.0%) 1 (6.2%) 1 (6.2%) 5 (31.2%) 2 (12.5%)	1 (3.8%) 9 (34.6%) 1 (3.8%) 5 (19.2%) 2 (7.7%) 8 (30.8%)
Pre-XRT DLQI n* = 33	None Small Moderate Large Very Large Extremely Large	4 (12.1%) 6 (18.2%) 10 (30.3%) 0 6 (18.2%) 7 (21.2%) *p = 0.638	1 (8.3%) 4 (33.3%) 3 (25.0%) 0 2 (16.7%) 2 (16.7%)	3 (14.3%) 2 (9.5%) 7 (33.3%) 0 4 (19.0%) 5 (23.8%)

LCT: Large cell transformation. XRT: treatment with radiation therapy. DLQI: Dermatology life quality index. *Fisher’s Exact (LCT vs. Non-LCT patients). ** T-Staging available for *n*=42 of the 46 patients, with 2a and 2b distinction only available for 8 of the 21 patients total with stage T2 disease

There is no significant difference in race, gender or age at presentation between patients with and without LCT (Table 1). However, patients with LCT presented at a higher T-stage prior to treatment, with 50.1% (9/18) presenting with greater than T2A disease compared to 39.3% (11/28) without LCT (*p*=0.001 by Fisher’s) (Table 1). The overall response rate for the entire cohort was 91.3%, 19.6% (9/46) had CR, 19.6% (9/46) had nearCR, 52.2% (24/46) had PR, and 8.7% (4/46) had SD. There was significantly better initial response to LD-TSEBT for patients with LCT compared to without. For patients with LCT, 38.9% (7/18) had CR, 22.2% (4/18) had nearCR, 38.9% (7/18) had PR, and 0% had SD, compared to 7.1% (2/28) with CR, 17.9% (5/28) with nearCR, 60.7% (17/28) with PR, and 14.3% (4/28) with SD for patients without LCT (*p*=0.028 by Fischer’s) (Table 2).

**Table 2:** Treatment response outcomes

		All Patients (*n*=46; 100%)	LCT Present (*n*=18; 39.1%)	LCT Absent (*n*=28; 60.9%)
Best Response	CR Near CR PR SD	9 (19.6%) 9 (19.6%) 24 (52.2%) 4 (8.7%)	7 (38.9%) 4 (22.2%) 7 (38.9%) 0	2 (7.1%) 5 (17.9%) 17 (60.7%) 4 (14.3%) *p*=0.028
Post-XRT Disease Stage n* = 45	1 A: n (%) 1B: n (%) 2 A: n (%) 2B: n (%) 3 A: n (%) 3B: n (%) 4 A: n (%) 4B: n (%)	28 (62.2%) 12 (26.7%) 1 (2.2%) 1 (2.2%) 1 (2.2%) 0 (2.2%) 1 (2.2%) 1 (2.2%) * ***p*** **=0.012**	14 (77.8%) 1 (5.6%) 0 1 (5.6%) 0 0 1 (5.6%) 1 (5.6%)	14 (51.9%) 11 (40.7%) 1 (3.7%) 0 1 (3.7%) 0 0 0
Post-XRT DLQI n* = 40	None Small Moderate Large Very Large Extremely Large	15 (37.5%) 11 (27.5%) 4 (10.0%) 1 (2.5%) 9 (22.5%) 0 *p=0.089	9 (56.2%) 4 (25.0%) 1 (6.2%) 1 (6.2%) 1 (6.2%) 0	6 (25.0%) 7 (29.2%) 3 (12.5%) 0 8 (33.3%) 0

CT: Large cell transformation. XRT: treatment with radiation therapy. DLQI: Dermatology life quality index. *Fisher’s Exact (LCT vs. Non-LCT patients)

**Fig. 1:**
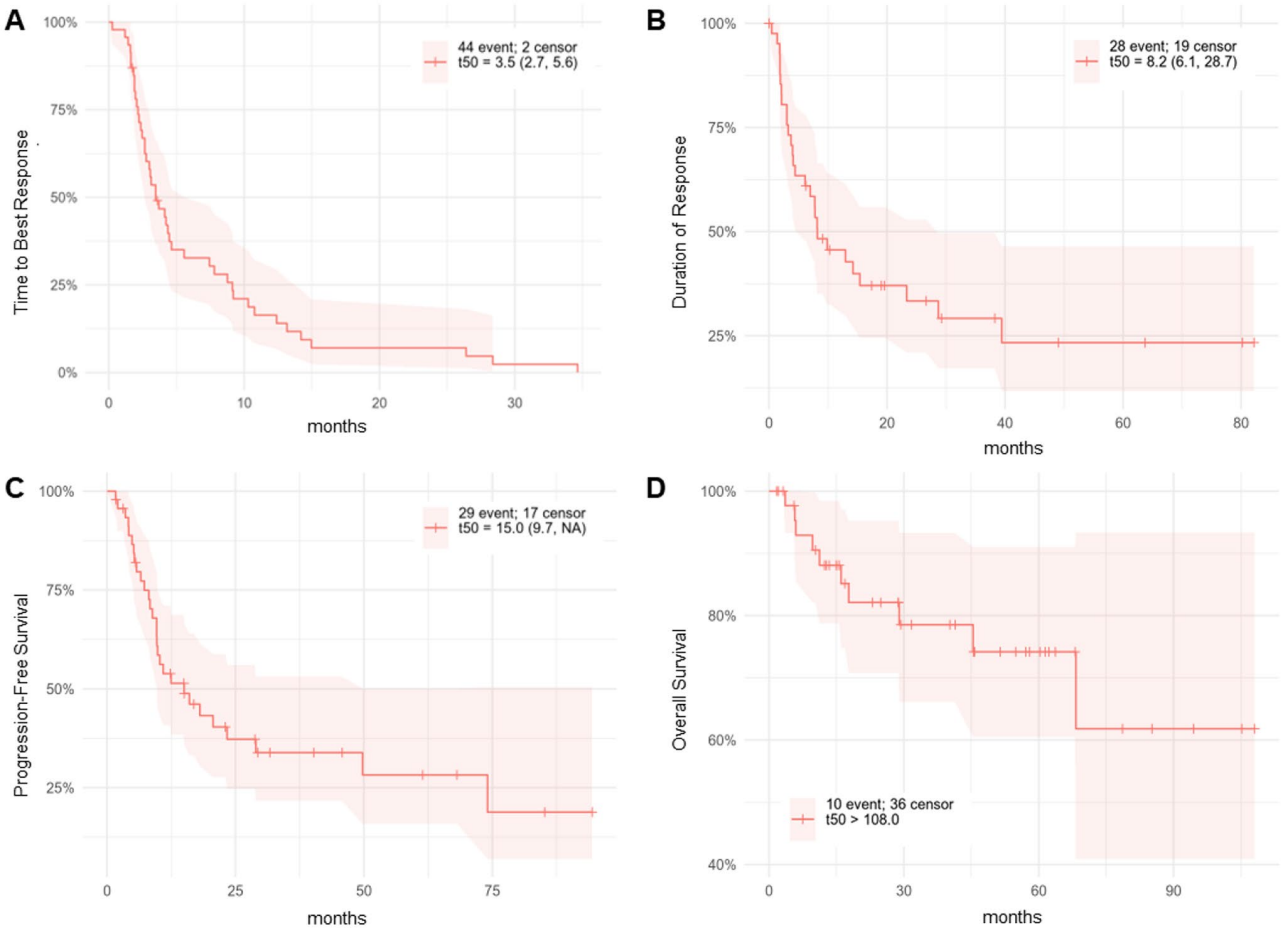
Treatment response over time for all patients. Kaplan-Meier analysis of (**A**) time to best response, (**B**) duration of response, (**C**) progression-free survival, and (**D**) overall survival for all patients after treatment

For the entire cohort, the median DTR was 8.2 months (range, 6.1–28.7), median TBR was 3.5 months (range, 2.7–5.6), median PFS was 15.0 months overall and OS was 75% at 5 years (Fig. 1A and D).

**Fig. 2:**
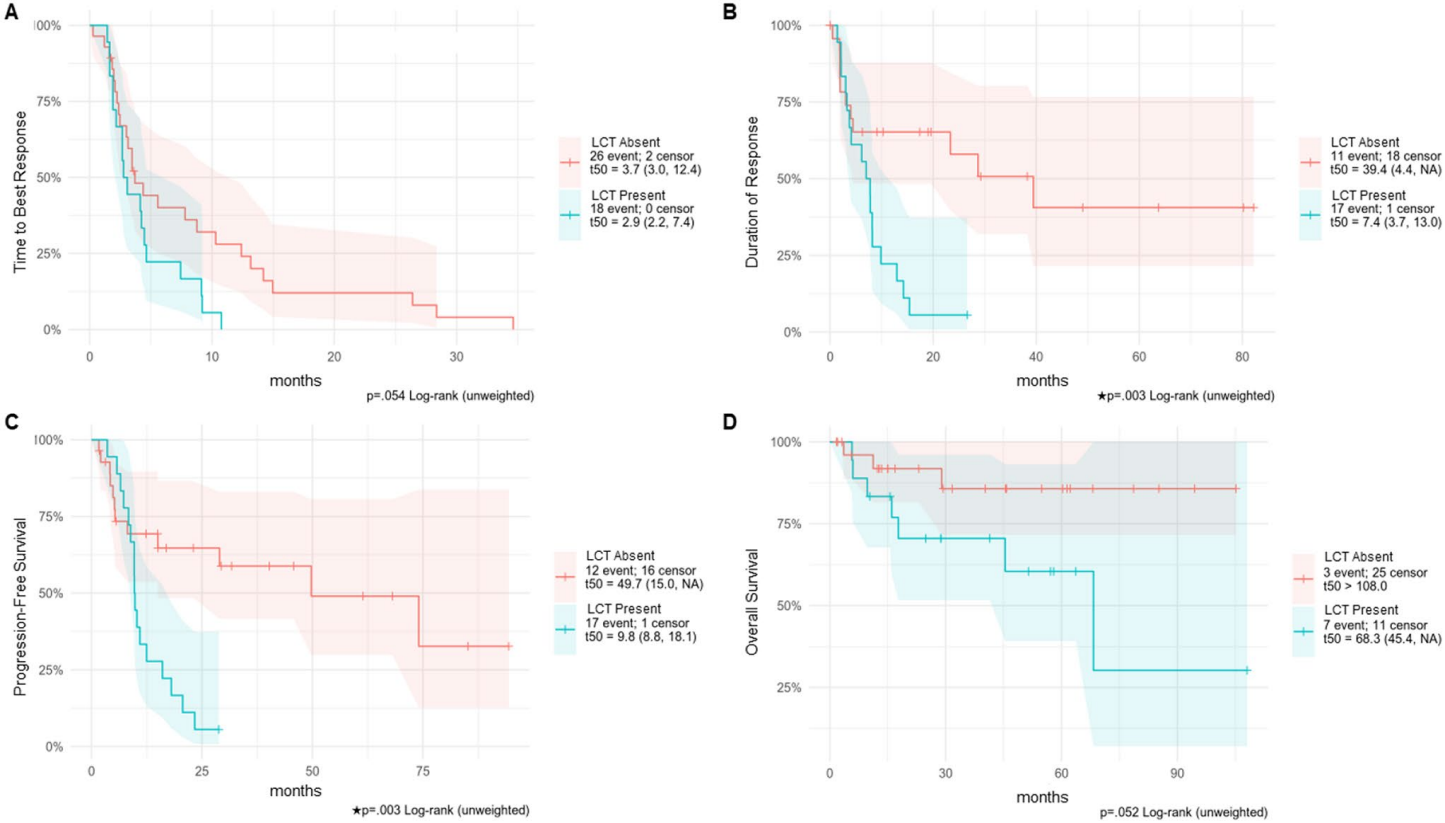
Treatment response over time stratified by LCT presence. Kaplan-Meier analysis comparing LCT and non-LCT patients by (**A**) time to best response, (**B**) duration of response, (**C**) progression-free survival, and (**D**) overall survival after treatment

Patients with LCT had a trend toward shorter median TBR at 2.9 months compared to the non-LCT cohort at 3.7 months, though not statistically significant (*p*=0.054). Median DTR and PFS were significantly lower in the LCT group compared to the non-LCT group at 7.4 versus 39.4 months (*p*=0.003) and 9.8 versus 49.7 months (*p*=0.003), respectively. Median OS was 68.3 months for patients with LCT versus >108 months for those without LCT (*p*=0.052) (Fig. 2A and D).

In regard to DLQI scoring, patients on average showed an improvement of 4.96 points (CI 2.13–7.80, *p*=0.0003). There was no statistical difference in pre-treatment DLQI score between the LCT and non-LCT (Table 1) and no difference in the change in mean score in the LCT and non- LCT cohort at 5.63 and 4.60 respectively (*p*=0.69).

### Toxicities

Toxicities related to TSEBT-related toxicities were minimal and reversible. Eleven patients experienced no adverse events (AEs) There were no grade 3 or higher AEs. The remaining thirty-five patients had either grade 1 or 2 toxicities, that all resolved within a few weeks to months after TSEBT completion. The spectrum of grade 1 and 2 toxicities included radiation dermatitis, fatigue, alopecia, nausea, vomiting, diarrhea, upper and lower extremity edema, pruritus, urticaria, and skin hyperpigmentation. The most common toxicity was grade 1 fatigue (*n*=13), grade 1 radiation dermatitis (*n*=12) and grade 1 alopecia (*n*=11). Six patients noted grade 2 radiation dermatitis, all of which self-resolved. Two patients did experience fingernail avulsion requiring a dose reduction to 10 Gy. No long-term AEs were observed.

## Discussion

This study further demonstrated that LD-TSEBT delivered at 12 Gy in six 2 Gy fractions every other day is effective for managing MF patients, with 91.3% of patients experiencing at least partial response to therapy. Our findings are consistent with a recent meta-analysis which found an overall response rate of 85% for LD-TSEBT (10–12 Gy) in CTCL [8, 10]. In comparison, high-dose radiotherapy of 30–36 Gy may offer an overall response rate of 99%, but at the cost of increased frequency and severity of side-effects and reduced scope for multiple re-treatments if necessitated [1, 8, 10]. The CR rate in our study was 19.6%, which is comparable to 28% as reported in other low-dose studies, but lower than 61% cited in the literature for high-dose [10]. However, the EORTC has noted that given the initial recommendations for high-dose therapy were made without comparative trials, a research gap exists for the optimal treatment dose [11, 12]. This in conjunction with long-term follow-up data from Standford showing increased disease recurrence over time, which reinforces the notion that modern treatment paradigms for MF should consider MF a chronic disease [6].

Half of the population in our study survived over 15 months without disease progression. Maintenace therapy following LD-TSEBT can include a topical nitrogen mustard, which has been proven to improve patient’s symptoms and delay disease progression [13, 14]. In addition, Phototherapy is another viable option for maintenance therapy after TSEBT for MF. The United States Cutaneous Lymphoma Consortium guidelines recommend phototherapy, specifically PUVA (psoralen plus ultraviolet A) and narrowband UVB (NB-UVB), as effective maintenance strategies [15]. Oral bexarotene has also been proved to be viable maintenance therapy following TSEBT improving response rates and PFS [16]. In the event of relapse, the benefit of LD-TSEBT is repeatability, given its low toxicity profile. This is a major advantage compared to conventional high-dose radiotherapy, in which fears of skin necrosis and bone marrow suppression preclude the ability to repeat treatment [1, 3].

We also identified that half of patients reached their best response within 3.5 months, achieved over 8 months of treatment response, and survived at least 9 years post-treatment. In comparison, a study of very low-dose therapy at 4 Gy identified a response duration of only 2.4 months, prompting a premature discontinuation of the study [12]. Patients in our study also displayed clinical improvement measured by an average 4.96 point increase in DLQI score. Balancing treatment tolerability with long-term disease control points toward low-dose TSEBT for the management of MF.

Within LD-TSEBT, optimal fractionation remains a question for radiation oncologists. A study of ultra-hypofractionated LD-TSEBT (8 Gy in two fractions) similarly found an 88.9% overall response rate, but the median PFS was 8 months and time to next treatment was 12 months [17]. In comparison, the fractionation scheme in our study (12 Gy in six fractions) seems preferable, given the median PFS of 15 months. However, our methodology does require patients to undergo treatment every other day for approximately two weeks.

A novel parameter tabulated in our study was “nearCR” or a near complete response. We believe in the utility of including nearCR as an option for categorizing treatment response, since its clinical significance is distinct from CR and PR. We believe this presents a more nuanced characterization of patient treatment response compared to other studies which tend to stratify by CR and overall response instead.

The response of the LCT cohort proved statistically and clinically distinct compared to non-LCT patients. It had been thought that LCT renders a poorer prognosis and an inferior response to TSEBT [4]. Interestingly, while the LCT cohort tended to present with more advanced disease (50.1% vs. 39.3% with high-staged MF) and shorter DTR these patients also had a more favorable initial response with superior rates of CR and nearCR at 61% (11/18) and 25% (7/28) respectively. The data would seem to characterize MF with LCT as an entity that, while aggressive, remains sensitive to radiation. This overall characterization should be considered when prescribing treatment for patients with MF and LCT, who may require dose escalation to achieve more sustained response times.

A limitation in this study is its retrospective nature, however given the rare nature of MF as an entity it is a challenge to design prospective studies. TSEBT is only offered at a few large centers worldwide, and meaningful prospective research will have to be multi-institutional in nature. Additionally, our analysis did not consider previous treatments, which have the potential to impact the disease response to radiation treatment. Small sample size limits the generalizability of our results as well.

The impact on patient reported quality of life for those undergoing multiple treatments after shorter DTR with LD-TSEBT versus those enjoying a longer DTR following HD-TSEBT remains a topic needing further study, although one prior study reported comparable outcomes [18]. Future research should be conducted longitudinally comparing the effect of several low-dose treatment regimens against HD-TSEBT on patient satisfaction and treatment toxicity to help guide optimal treatment strategies.

In conclusion, our findings support the use of condensed LD-TSEBT as a convenient treatment option for MF, offering favorable clinical outcomes and a low toxicity profile. Our results also indicate that patients with LCT treated with LD-TSEBT experience faster improvements in disease status, but shorter durations of treatment response, compared to those without LCT. Further studies are warranted to verify this finding and establish strategies to prolong the duration of response.

## Data Availability

No datasets were generated or analysed during the current study.
